# Clinical Course and Mortality Predictors in Adult Hospitalized Patients with COVID-19 Infection—A Retrospective Cohort Study

**DOI:** 10.3390/medicina61040579

**Published:** 2025-03-24

**Authors:** Vesela Blagoeva, Vladimir Hodzhev, Petar Uchikov, Bistra Dobreva-Yatseva, Rumyana Stoyanova, Maritza Shterev, Samiya Atiq, Akanksha Prasad, Sriharini Shankar Babu

**Affiliations:** 1 Pulmonology and Tuberculosis Section, First Department of Internal Medicine, Medical University of Plovdiv, 4002 Plovdiv, Bulgaria; vhodzhev@abv.bg; 2 Department of Special Surgery, Medical University of Plovdiv, 4002 Plovdiv, Bulgaria; petar.uchikov@mu-plovdiv.bg; 3 First Department of Internal Medicine, Cardiology Section, Medical University of Plovdiv, 4002 Plovdiv, Bulgaria; bistra0806@yahoo.com; 4 Department of Health Management and Health Economics, Medical University of Plovdiv, 4002 Plovdiv, Bulgaria; rumyana.stoyanova@mu-plovdiv.bg; 5 Medical Faculty, Medical University of Plovdiv, 4002 Plovdiv, Bulgaria; maritzachterev@gmail.com (M.S.); samiya.atiq99@gmail.com (S.A.); sbsriharini@gmail.com (S.S.B.)

**Keywords:** mortality predictors, COVID-19 infection, hospitalized patients, severity

## Abstract

*Background and Objectives*: Bulgaria had the highest mortality rate of COVID-19 in Europe and the second highest in the world based on statistical data. This study aimed to determine the mortality predictors in 306 adult patients with COVID-19 infection, treated at the COVID-19 Ward of St. George University Hospital in Plovdiv, Bulgaria in the period of August 2021–April 2022. *Materials and Methods*: All admitted and treated patients had a positive PCR test for SARS-CoV-2. They were assigned in three groups based on the severity rating scale published in NIH COVID-19 Treatment Guidelines by Stat Pearls Publishing, 2022. Demographic, clinical, and laboratory parameters and pre-existing comorbidities were investigated. Parametric and non-parametric methods were used for statistics. Logistic regression was applied for parameters significantly associated with mortality. *Results*: Data showed that demographic indicators were not significantly associated with poorer outcome. Among comorbidities, cardiovascular, chronic pulmonary and endocrine disorders were found to be related to poor survival rates (*p* = 0.003, *p* = 0.003 and *p* = 0.017 resp.) Clinical symptoms, such as sore throat, dry or productive cough and breathlessness, were determinants of poor outcome (*p* = 0.027, *p* = 0.029, *p* = 0.004 and *p* = 0.002 resp.). Laboratory parameters linked to mortality were elevated d-dimers (*p* = 0.015), ferritin (*p* = 0.009) and creatinine (*p* = 0.038). p02 < 50 and saturation < 90 also indicated a higher risk of death (*p* = 0.006 and *p* = 0021). *Conclusions*: Logistic regression showed that each stage of disease severity increased the risk of death 3.6 times, chronic pulmonary disorders increased it by 4.1, endocrine by 2.4 and dyspnea by 3.1 times.

## 1. Introduction

The global toll of COVID-19 has claimed over 7 million lives, and new cases are still being registered [1].

Bulgaria was among the countries most severely affected by the pandemic. Based on data from the WHO, by 15 November 2022, there were 1,284,051 confirmed COVID-19 cases in all 28 regions of the country, with 37,965 being lethal cases [1]. Bulgaria ranks 87 worldwide regarding the number of infected per 100,000 of the population but second after Peru and first in Europe in terms of mortality rate. According to data from the John Hopkins Institute, the COVID-19 mortality rate in Bulgaria was 550/100,000 [2]. Moreover, Bulgaria had the highest excess mortality rate in Europe during the first year of the pandemic—approximately 27%, which was nearly twice the median excess mortality rate of 14% in Europe [3]. The excess mortality rate in our country remained high until the end of the pandemic—10% compared to 6–8% in Europe, on average [3]. Additionally, research, conducted in Bulgaria and in Europe has showed that unlike other European countries, the excess mortality rate in Bulgaria was highest in the age group <65. On the contrary, for instance in Italy and in Spain, the mortality rate in this age group is only 7% and in Europe as whole, most lethal cases were registered in patients >80—67–70% of the cases. Various explanations for these statistics have been suggested: low level of testing among the population, non-compliance with the anti-epidemic measures, high incidence of comorbidities in the general population (especially cardiovascular), uneven distribution of medical facilities throughout the country, and finally, the low level of vaccine coverage—26.7% compared to 66.1% average in Europe [4].

At present, a vast number of research works and systematic reviews from all over the world (including Bulgaria) have investigated more than 70 different demographics [3,5,6], clinical [7], laboratory [8,9,10,11] and imaging factors [12] in an attempt to elucidate which of them increased the risk of lethal outcome in patients with COVID-19 infection in different settings. So far, they have documented controversial results.

The aim of the present study was to investigate a panel of demographic, clinical and laboratory parameters, as well as pre-existing comorbidities, that were associated with increased mortality in 306 adult hospitalized Bulgarian patients with COVID-19 infection.

## 2. Materials and Methods

Our study is a retrospective, observational, mono-centered study. It was conducted at the COVID-19 Ward based at the Clinics of Pulmonology of the St. George University Hospital in Plovdiv, Bulgaria. The Clinics of Pulmonology provide medical services to adult patients with acute and chronic respiratory disorders for the region of Plovdiv and South Bulgaria. During the pandemic, patients were referred to the Clinics either from the Hospital Emergency Department, from other hospital wards or from other hospitals in Plovdiv and the area after a positive professional-use antigen test. Only adult patients (>18 years old) presenting with symptoms consistent with COVID-19 (sudden onset of high fever, cough, dyspnea) were admitted to the clinics. On the day of admission, all of them additionally had a PCR test from a nasal–throat swab. PCR tests were performed using ExiStation equipment and Bioneer COVID-19 RT PCR kits (Yangsan-si, South Korea). Thus, all patients met the WHO criteria for a confirmed SARS-CoV-2 infection: positive PCR, regardless of the clinical and epidemiological data or clinical symptoms and/or epidemiological, and a positive Ag test [13]. Subsequently, patients were registered into the National Registry for COVID-19 cases and signed an informed consent. None of them had a history of previous infection with COVID-19. Out of all patients treated at the COVID-19 Ward during the period August 2021–April 2022, 306 were randomly selected for the study. For the purpose of analysis, they were divided into four groups: mild, moderate, severe, and critical illness (based on the severity rating scale as cited in Features, Evaluation and Treatment of Coronavirus [COVID-19], Stat Pearls Publishing; 2022 [14]). Individuals <18 years old, asymptomatic patients and those without a positive PCR test were excluded (Table 1).

Data were collected from the electronic hospital register and the patients’ electronic medical records. All requirements regarding confidentiality of medical and personal information were followed strictly according to the General Data Protection Regulations (GDPR, 2016/679) issued by the European Parliament (the dataset included prior anonymization of patients and no other personal identifiers). The relevant data included: demographic indicators, such as age, sex, place of residence, smoking history, and institutionalization, followed by a detailed clinical history and registration of all pre-existing comorbidities. Apart from the history, comorbidities diagnosed prior to infection with SARS-CoV-2 were confirmed based on the patients’ electronic records, available through their GPs’ documentation. The focus was vascular (CVD and cerebrovascular), malignant, chronic lung and endocrine disorders as they are the most common worldwide, including Bulgaria.

Based on the clinical history, symptoms were divided into: general: fever > 37 °C and malaise; upper respiratory tract: sore throat, nasal discharge, loss of olfaction and taste; lower respiratory tract: dry or productive cough, blood in the sputum, dyspnea; gastro-intestinal (GI) symptoms: appetite loss, nausea, vomiting, diarrhea, abdominal pain and mental state changes. Laboratory parameters investigated included: CBC with differential blood count, liver enzymes (AST ALT GGT), urea, creatinine, CRP, LDH, fibrinogen, ferritin, d-dimers, ABG (arterial) parameters as p02, and saturation. Rules for patient anonymity and confidentiality were strictly followed (prior anonymization and no personal identifiers).

### Statistical Analysis

The data were exported to IBM SPSS v. 23 statistical software and analyzed with descriptive statistics, parametric and non-parametric methods. Statistical analysis included descriptive methods for the qualitative and quantitative variables to determine absolute and relative frequencies. Parametric and non-parametric methods (Fisher’s exact test and chi-square test) were used to assess the statistical significance of the relevant parameters. Logistic regression analysis was used to assess the association between independent variables and mortality risk, with results reported as odds ratios (OR) and 95% confidence intervals (CI). The level of significance of 5% probability (*p* < 0.05) was adopted. The initial regression model included all variables, statistically correlated with morality by univariate analysis, i.e., *p* < 0.05 (Fisher’s exact test, chi-square test). The stepwise multivariate regression model was applied to control for potential confounders. Many of the variables in the initial model were excluded as not significant. The final model included only the significant variables.

## 3. Results

The study included 306 patients with COVID-19 infection, confirmed by the PCR test from a nasal–throat swab. Based on the disease severity scale, patients were divided into three groups: patients with moderate, 149 (48.7%); with severe, 121 (39.5%); and with critical infection, 23 (7.9%). The remaining 13 (3.9%) were classified as mild COVID-19. Out of 306 patients, 274 (89.5%) were discharged, while 32 (10.5%) had a lethal outcome. The median patients’ age was 67 ± 13.06, ranging from 26 to 92 years old. Male-to-female ratio was approximately equal: 146 men and 160 women (48% and 52%, respectively). Of these patients, 220 were urban residents (72%), and the remaining 86 (28%) village dwellers. Of them, 257 (84%) were non-smokers, whereas 49 (16%) were smokers. Only 12 (4%) of the patients were nursing home residents. The statistical analysis showed that in our cohort of in-patients, the age of those who had a lethal outcome was 70.6 years old; therefore, they were older compared to the age of discharged, 66.7. Similarly, the results regarding the sex–mortality rate was higher in men (11.9%) compared to in women (8.9%); however, the differences between both groups were not statistically significant regarding both studied variables. Smoking history, place of residence and institutionalization were not found to be related with a more severe course and mortality in COVID-19 infection (Table 2).

Regarding pre-existing underlying medical conditions, the comparison between patients with or without pre-existing comorbidities documented that the presence of underlying medical disorders increased the risk of poor outcome in COVID-19 infection (Table 3).

Analysis of the nature of comorbidities demonstrated that pre-existing cardiovascular, lung and endocrine disorders were associated with an increased risk of lethal outcome in COVID-19 infection; the difference was statistically significant, as seen in Table 4. Neurological conditions, malignancies and chronic renal disorders did not seem to significantly affect the outcome.

Another important focus in our study was the attempt to identify the clinical symptoms more typical of patients with COVID-19-infection and to register those of them that could be associated with an increased risk of death. To facilitate analysis of symptoms, they were divided into groups as follows: upper respiratory tract symptoms absent in 257 (84%) of the patients; of those that had upper respiratory tract symptoms, 36 (11.8%) had sore throat, 12 (3.9%) had nasal congestion and only one (0.3%) had loss of smell and taste. Lower respiratory tract symptoms were absent in 46 (16%) of the patients. The remaining 260 (86%) presented with dry cough, (181, 59.1%), productive cough (77, 25.2%), and 2 (0.7%) had bloody sputum. All patients were asked about the presence of dyspnea: present in 163 and absent in 143 patients (53.3% and 46.7%, respectively). Regarding gastro-intestinal symptoms, they were absent in 222 (72.5%) of the patients. Of the remaining, 71 (23.2%) had appetite loss and nausea, 11 (3.6%) had diarrhea, and 2 (0.7%) had diffuse abdominal pain. All above-mentioned symptoms were statistically analyzed to check their relation with higher risk of lethality in COVID-19 infection. Among the studied symptoms, those associated with higher risk of lethal outcome were sore throat, presence of dry or productive cough, and dyspnea. The results of the statistical analysis are presented in Table 5.

On day one of admission, all 306 inpatients had a panel of routine laboratory tests performed, including CBC, liver enzymes, urea and creatinine, CRP, fibrinogen and d-dimers. In terms of laboratory parameters, our data showed that elevated ferritin, creatinine and d-dimer levels were determinants of a higher risk of lethal outcome. Similarly, low saturation and low p02 level also indicated severe course and elevated risk for poor outcome. The results are presented in Table 6.

A regression model was applied to the variables that were significantly correlated with mortality. The results of the significance test were as follows: χ^2^ = 14.816, DF = 4, *p* = 0.063, *p* > 0.05, indicating that the model is optimal. Logistic regression showed that chronic lung disorders increase the risk of death by 4.136 times (OR = 4.136, 95% CI: 1.710–10.004, *p* = 0.002), followed by dyspnea, 3.179 (OR = 3.179, 95% CI: 1.326–7.625, *p* = 0.010) and endocrine disorders, 2.47 (OR = 2.471, 95% CI: 1.083–5.641, *p* = 0.032). Each level of severity also results in higher risk of lethal outcome, 3.697 times (OR = 3.697, 95% CI: 1.984–6.821, *p* = 0.000). The results are presented in Table 7. Table 8 depicts the correlation matrix of independent variables included in the regression model.

## 4. Discussion

The mortality rate in our patients was 10.5%. Data on the mortality rate of COVID-19 in hospitalized patients are controversial; however, a systematic review and meta-analysis from Portugal, including 33 studies (mostly from China, Thailand, USA, Spain and Italy), reports a mortality rate of 11.5% [15] for hospitalized patients, which is similar to that in our study. It is worth mentioning that the global mortality rate of COVID-19 was calculated at 6.73% [15]. The established higher mortality rate in our study was attributable to the inclusion of only hospitalized patients who represented the more severe subgroup of COVID-19 patients. Regarding the demographic indicators, we found that in our study they do not influence the risk of death in COVID-19 infection. The mean age of recovered patients was 66.7 years and that of diseased was 70. 6 years, the difference being not statistically significant. On the contrary, evidence from the available literature shows a definite trend of increased mortality in older patients. Data from the USA indicate that 62% of patients hospitalized for COVID-19 were >55 years of age [16], whereas less than 1% of them were < 19 years of age [16]. Similarly, CDC data show a tendency of increased mortality with age—in the age group 54–60, it is 17.3%; in the age group 65–74, 22.18%; and in the age group >75, the mortality rate is 54.14% [17]. Studies from China document the same tendencies—the median age of discharged patients was 56.0 ± 13.5 and that of diseased, 70.2 ± 7.7, the difference being statistically significant (*p* = 0.001). In Italy, in a study of 355 patients, the median age of diseased patients was 79.5 [9,18]. The reasons for the increased mortality with age in COVID-19 infection are not yet fully elucidated [19]. However, the most likely explanation are the changes in the immune system with age and the more common comorbidities in this age group [20]. Unlike other European countries, in Bulgaria, analysis of the pandemic demonstrates a number of specifics: firstly, there is a high percentage (28%) of lethal cases in the working age group 45–65 [4]. A study by Raganchev et al. [4] explains this by the very high incidence of CVD in Bulgaria within this age group. Secondly, the same authors found that there were a high number of outbreaks at workplaces, especially in garment, textile, battery and automobile factories. Therefore, the higher percentage of work age patients with COVID-19 and the high incidence of CVD are a likely explanation for our findings. Moreover, in our study, the small percentage of nursing home residents (only 3.9%) and the small percentage of vaccination coverage in Bulgaria (−33% versus 66% in Europe, on average) seems to have influenced the results: a slightly higher age of diseased patients without statistically significant differences between the age groups. In this study, male-to-female patient ratio was similar with a mild predominance of male patients: 47% versus 52%. Many research studies so far have shown that the male gender is a risk factor for mortality [18,21], and 60% of deaths are male patients aged at least 60 years. It has been speculated that generally women are capable of building a stronger immune response than males, and there are data on the protective effect of estrogen exposure against COVID-19 infection [9]. However, based on our data, in moderate, severe and critical cases of COVID-19, mortality rates do not differ significantly in both sexes. Similar findings were published in Italy: in more severe forms of infection, especially in patients needing NIV or invasive ventilation, the mortality rate is similar in both sexes [19]. In terms of comorbidities, our data are consistent with that available in the literature, reporting increased mortality rates in patients with comorbidities. As seen from Table 4, the presence of at least one comorbidity increases significantly the risk of lethal outcome. It is considered that approximately half of the lethal cases are either of patients with CVD or diabetes. Multiple research studies have provided evidence that comorbidities increase the risk, and among them, CVD are ranked first, followed by diabetes and chronic lung disorders [20]. Endothelial dysfunction is one of the very first changes in CVD and diabetes. There are data showing that the SARS-CoV-2 virus binds to the ACE2 receptor in the endothelial cells [18], thus causing vasculitis. Myocardial damage via direct viral invasion and hypoxia have also been suggested to play a role [5]. Regarding diabetes, apart from the endothelial damage, the study by Muniyappa R and Gubbi S have suggested several other mechanisms: hyperglycemia induced inhibition of intracellular destruction of microbes, neutrophil chemotaxis, and phagocytosis providing higher affinity for cellular binding and effective virus entry, and reducing viral clearance [7,22] Furthermore, it is believed that SARS-CoV-2 uses ACE2 as entry receptors. These receptors are present on the islets of Langerhans. A research study from China has put forward the probability of mild to fulminant damage to these cells, leading to clinical states varying from mild hyperglycemia to lifethreatening diabetic ketoacidosis [23].

The role of chronic pulmonary disorders and their impact on survival in COVID-19 seems less discussed in the literature. In our study, we found that chronic lung disorders (mostly COPD) increased the risk of death. In our study, all asthmatic patients survived. Similar data are present in other studies, showing no increased risk in asthma and even less severe course of COVID-19 in these patients. Possible mechanisms include decreased ACE2 receptor expression that lowers the risk of COVID-19 severity [24]. It has also been suggested that T helper 2 (Th2) immune response in patients with asthma may counter the inflammation induced by SARS-CoV-2 infection [14]. On the contrary, COPD patients seem prone to worse outcome in COVID-19 infection [25]. A systematic review [25] showed increased mortality in these patients, 17%. Moreover, the ACE2 receptor again seems to play a role as there is evidence of its increased expression in the respiratory epithelial cells in COPD. Additionally, COPD patients are at an older age, are more prone to clot formation, and often have other comorbidities as CVD [25].

The clinical presentation of COVID-19 has a wide spectrum that ranges from asymptomatic or paucisymptomatic forms to the development of bilateral pneumonia, respiratory failure, respiratory distress and death. In this study, the presence of upper respiratory tract symptoms had no impact on mortality rates. This is in accordance with other research works, which report that these symptoms are common in mild forms of COVID-19, but their incidence in hospitalized patients in very low, 4% [25,26,27]. On the contrary, there is evidence that lower respiratory tract symptoms, such as dry or productive cough and especially the presence of dyspnea, are more prevalent in diseased patients compared to survivors. Dyspnea is a known risk factor for mortality based on a systematic review from China [28]. Moreover, a study from Italy associates cough and dyspnea with pneumonia and shows that they are determinants of severe course and higher mortality rates [29]. Regarding the presence of sore throat, evidence is controversial; some studies support its role as predictor of increased risk of severe course and death [26,30]. However, most studies report that upper respiratory tract symptoms (loss of olfaction, nasal discharge, sore throat, etc.) and GI symptoms, such as vomiting and diarrhea, were not associated with increased risk of death. There is evidence in the literature that even they are associated with milder course and better recovery rates [31].

Finally, in terms of the laboratory parameters, our study documented that elevated levels of d-dimers (>0.5 mg/L), ferritin (>150 µmol/L) and creatinine (>97 µmol/L) are related with poorer outcomes in COVID-19 infection. A large number of research from all over the world indicate consistently that high d-dimers are associated with increased risk of mortality [10,12]. The likely explanation is the endothelial cell dysfunction caused by the virus, as well as the systemic pro-inflammatory cytokine response. Regarding CRP, LDH and high creatinine, available evidence for their role of determinants of prognosis is not sufficient. Studies from China in 1099 patients from 552 hospitals in 30 provinces documented that high levels of LDH and CRP were also associated with severe course of the disease and high mortality rates [12,32,33,34]. Another study, also from China, found an association between CRP, LDH, troponin, creatinine, and serum albumin as predictors of mortality [8]. There is evidence for the role of increased creatinine level as related to poorer outcome in another study from China [18]. Regarding ferritin level, data in the literature seem limited; however, the same study from China in 817 in-patients confirmed its role as related to increased risk of death [18].

*Study limitations*. The main limitations of this study are: the mono center character of the study and the relatively limited number of patients, involved. Another limitation is the retrospective character; which potentially implies missing data and lack of patient follow-up, therefore the long-term consequences of COVID-19 infection were not assessed.

The inclusion of only adult hospitalized patients; exclusion of patients with asymptomatic infection; inclusion of very few patients with mild infection and exclusion of the pediatric population do not allow extrapolation of the results to the general population.

## 5. Conclusions

Based on our findings, comorbidities, especially CVD, chronic pulmonary disorders (mostly COPD) and diabetes are associated with poorer outcomes in COVID-19 infection. Among clinical symptoms, cough and shortness of breath are related to disease severity and death. Elevated d-dimers, creatinine and ferritin are laboratory markers, indicating a higher risk of death. Moreover, each stage of disease severity increased the risk of death 3.6 times, chronic pulmonary disorders increased it by 4.1, endocrine by 2.4 and dyspnea by 3.1 times. Better knowledge of these risk factors contributes to better patient stratification on admission, and further studies will improve the outcome.

## Figures and Tables

**Table 1:** Severity rating scale for COVID-19 infection (updated March 2023).

Asymptomatic and presymptomatic infection	Asymptomatic individuals with a positive PCR for SARS-CoV-2 without any clinical symptoms consistent with COVID-19
Mild illness	Individuals who have symptoms of COVID-19, such as fever, cough, sore throat, malaise, headache, muscle pain, nausea, vomiting, diarrhea, anosmia, or dysgeusia but without shortness of breath or abnormal chest imaging
Moderate illness	Individuals who have clinical symptoms or radiologic evidence of lower respiratory tract disease and who have oxygen saturation (SpO_2_) ≥ 94% at room air
Severe illness	Individuals who have SpO_2_ ≤ 94% at room air, a ratio of partial pressure of arterial oxygen to fraction of inspired oxygen, (PaO_2_/FiO_2_) of less than 300, with marked tachypnea with respiratory frequency > 30 breaths/min or lung infiltrates > 50%
Critical illness	Individuals who have acute respiratory failure, septic shock, and/or multiple organ dysfunction. Patients with severe COVID-19 illness may become critically ill with the development of acute respiratory distress syndrome (ARDS), which tends to occur approximately one week after the onset of symptoms

**Table 2:** Demographic indicators and relationship with disease severity and outcome.

Demographic Characteristics		Total Number of Patients n = 306 (100%)	Survivors n = 274 (89.5%)	Non-Survivors n = 32 (10.5%)	*p*
Age (Mean ± SD)		67.1 ± 13.6	66.7	70.6	0.125
Sex	Men	160 (52.5%)	141 (88.1%)	19 (11.9%)	0.457
	Women	146 (47.5%)	133 (91.1%)	13 (8.9%)	
Smoking	Non-smokers	257 (84%)	232 (89.5%)	25(10.5%)	0.234
	Smokers	49 (16%)	42 (85.7%)	7 (14.3%)	
Residence	Town	220 (71.9%)	193 (87.7%)	27 (12.3%)	0.680
	Village	86 (28.1%)	81 (92.4%)	5 (5.8) %)	
Institutionalization	No	294 (96.1%)	262 (89.1%)	32 (10.9%)	0.259
	Yes	12 (3.9%)	12 (100%)	0 (0%)	

**Table 3:** Presence or absence of comorbidities and relationship with disease severity and outcome.

Characteristics	Number of Patients n (%)	Survivors n (%)	Non-Survivors n (%)	*p*
No comorbidities	44 (14.3%)	43 (97.7%)	1 (2.3%)	0.001 *
With pre-existing comorbidities	262 (85.7%)	231 (88.1%)	31 (11.9%)	

* Significance differences. Only statistically significant *p* appear in bold.

**Table 4:** Nature of comorbidities and relationship with disease severity and outcome.

Nature of Comorbidities		Number of Patients n (%)	Survivors n (%)	Non-Survivors n (%)	*p*
Cardio-vascular disorders	No	94 (30.7%)	91 (96.8%)	3 (3.2%)	0.003 *
	Yes	212 (69.3%)	183 (86.3%)	29(13.7%)	
Chronic lung disorders	No	254 (30.7%)	242 (92.1%)	20 (7.9%)	0.003 *
	Yes	52 (69.3%)	40 (76.9%)	12 (29.1%)	
Chronic renal disorders	No	286 (93.5%)	256 (89.5%)	30 (11.5%)	0.561
	Yes	20 (9.5%)	18 (90%)	2 (10%)	
Malignancies	No	271 (88.6%)	243 (89.7%)	28 (10.3%)	0.513
	Yes	35 (11.4)	31 (88.6%)	4 (11.4%)	
Neurological disorders	No	263 (85.9%)	236 (89.7%)	27 (10.3%)	0.478
	Yes	43 (14.1%)	38 (88.4%)	5 (11.6%)	
Endocrine disorders	No	226(73.9%)	208 (92%)	18 (8.0%)	0.017 *
	Yes	80 (26.1%)	66 (82.5%)	14 (17.5%)	

* Significant differences. Only statistically significant *p* appear in bold.

**Table 5:** Clinical symptoms and laboratory markers associated with poor outcome.

Symptoms		Total Number n = 306 (100%)	Survivors n = 274 (89.5%)	Non-Survivors n = 32 (10.5%)	*p*
Fever > 37 °C	No	89 (29%)	79 (88.8%)	10 (22.2%)	0.459
	Yes	217 (71%)	195 (89.9%)	22 (10.1%)	
Sore throat	No	257 (83.9%)	232 (90.3%)	25 (9.7%)	0.027 *
	Yes	36 (16.1%)	31(86.1%)	5 (13.9%)	
Rhinitis	No	294 (96%)	232 (90.3%)	25 (9.7%)	0.213
	Yes	12 (4%)	11(91.7%)	1 (8.3%)	
Dry cough	No	46 (15.0%)	46 (100%)	0	0.029 *
	Yes	Yes—181 (59.15)	162 (89.5%)	19 (10.5%)	
Productive cough	No	46 (15%)	46 (1005)	0	0.004 *
	Yes	77 (25.1%)	64 (83.1%)	13 (16.9%)	
Dyspnea	No	163 (53.3%)	154 (94.5%)	9 (5.5%)	0.002 *
	Yes	143 (47.7%)	120 (83.9%)	23 (16.1)	
Mental state changes	No	281 (91.8%)	254 (90.6%)	27 (9.6%%)	0.104
	Yes	25 (8.2%)	20 (80%)	5 (20%)	
Nausea	No	222 (72.5%)	197 (88.7%)	25 (11.3%)	0.384
	Yes	71 (23.2%)	64(90.1%)	7 (9.9%)	
Diarrhea	No	222 (72.5%)	197 (88.7%)	25 (11.3%)	0.375
	Yes	11 (3.6%)	11 (100%)	0	

* Significant differences. Statistically significant *p* are in bold.

**Table 6:** Laboratory parameters and relationships with severe course and lethality.

Laboratory Parameters		Number of Patients n (%)	Survivors n (%)	Non-Survivors n (%)	*p*
Haemoglobin Hg < 140 g/L (female) Hg < 160 g/L (male)		271(88.6%)	242 (89.3%)	29 (10.7%)	0.487
		35(11.4%)	32 (91.4%)	3 (8.6%)	
Leucocytes: 4–10 g/L >10—leucopenia >4—leucopenia		177 (91.2%)	17 (8.8%)	17 (8.8%)	0.131
		67 (83.8%)	67 (83.8%)	13 (16.3%)	
		30 (93.8%)	30 (93.8%)	2 (6.3%)	
CRP > 10 g/L	≤10	31 (89.8%)	29 (93.6%)	2 (6.5%)	0.334
	>11	275 (10.1%)	245 (89.1%)	30 (10.9%)	
LDH > 460 U/L	≤460	92 (30.0%)	84 (91.3%)	8 (8.7%)	0.331
	>460	214 (70%)	190 (88.2%)	24 (20.2%)	
Ferritin > 150 µmol/L	≤150	31 (10.1%)	56 (92.8%)	1 (1.8%)	0.009 *
	>150	275 (89.9%)	218 (87.6%)	31 (12.4%)	
ASAT > 35 U/L	<35	135 (44.1%)	120 (88.9%)	15 (11.1%)	0.397
	>35	171 (55.8%)	146 (85.4%)	25 (14.6%)	
ALAT > 36 U/L	<36	165 (53.9%)	142 (86.7%)	23 (13.3%)	0.511
	>36	141 (47.1%)	143 (88.7%)	18 (12.8%)	
GGT > 38 U/L	<38	127 (41.5%)	114 (89.8%)	13 (10.2%)	0.233
	>38	179 (58.5%)	152 (84.9%)	27 (15.1%)	
Creatinine µmol/L	≤97	228 (10.1%)	209 (91.4%)	19 (8.6%)	0.038 *
	>97	78 (89.9%)	65 (84.7%)	13 (15.3)	
Urea mmol/L	≤8	234 (76.4%)	212 (90.6%)	22(9.4%)	0.191
	>8	72 (24.6%)	62 (86.1%)	10 (13.9%)	
D-Dimers mg/L	≤0.5	103 (33.6%)	98 (95.1%)	5 (5.1%)	0.015 *
	>0.5	203 (66.5%)	176 (86.7%)	27 (13.3%)	
Fibriogen g/L	≤4.5	132 (43.1%)	123 (92.3%)	9 (6.8%)	0.036
	>4.5	174 (56.9%)	151 (86.8%)	23 13.2%)	
pO_2_ mmHg	>50	230 (75.1%)	216 (92.3%)	14 (7.7%)	0.006 *
	≤50	76 (24.8%)	58 (80.6%)	18 (19.4%)	
s0_2_%	>90	207 (67.4%)	182 (89.4%)	22 (10.6%)	0.001 *
	<90	99 (32.6%)	81 (81.8%)	18 (18.2%)	

* Significant differences. Statistically significant *p* are in bold.

**Table 7:** Regression analysis of parameters correlated with mortality.

Variables in the Equation									
		B	S.E.	Wald	df	Sig.	Exp(B)	95% C.I. for EXP(B)	
								Lower	Upper
Step 1 ^a^	Dyspnea	1.157	0.446	6.718	1	0.010	3.179	1.326	7.625
	Severity	1.303	0.315	17.101	1	0.000	3.679	1.984	6.821
	Endocrine disorders	0.905	0.421	4.618	1	0.032	2.471	1.083	5.641
	Lung disorders	1.420	0.451	9.927	1	0.002	4.136	1.710	10.004
	Constant	−7.074	1.079	42.954	1	0.000	0.001		

^a^ Variable(s) entered in step 1: dyspnea, severity, endocrine, lung disorders.

**Table 8:** Correlation matrix of independent variables in the logistic regression model.

		Constant	Dyspnea	Severity	Endocrine Disorders	Lung Disorders
Step 1	Constant	1.000	−0.395	−0.914	−0.196	−0.295
	Dyspnea	−0.395	1.000	0.120	0.062	−0.010
	Severity	−0.914	0.120	1.000	0.008	0.174
	Endocrine disorders	−0.196	0.062	0.008	1.000	0.107
	Lung disorders	−0.295	−0.010	0.174	0.107	1.000

Note: None of the independent variables have high correlations (r > 0.8), meaning multi-collinearity is not a problem and no need to remove any variables from regression model.

## Data Availability

Restrictions apply to the availability of these data. Data were obtained from the electronic hospital database and are available from the authors with permission from the St. George Hospital Administration.
